# Inflammaging and Frailty in Immune-Mediated Rheumatic Diseases: How to Address and Score the Issue

**DOI:** 10.1007/s12016-022-08943-z

**Published:** 2022-05-21

**Authors:** Fausto Salaffi, Andrea Di Matteo, Sonia Farah, Marco Di Carlo

**Affiliations:** Rheumatology Clinic, Università Politecnica Delle Marche, Carlo Urbani” Hospital, Via Aldo Moro, 25, 60035 Jesi Ancona, Italy

**Keywords:** Immune-mediated rheumatic diseases, Frailty, Inflammaging, Sarcopenia, Ageing population

## Abstract

Frailty is a new concept in rheumatology that can help identify people more likely to have less favorable outcomes. Sarcopenia and inflammaging can be regarded as the biological foundations of physical frailty. Frailty is becoming more widely accepted as an indicator of ageing and is linked to an increased risk of negative outcomes such as falls, injuries, and mortality. Frailty identifies a group of older adults that seem poorer and more fragile than their age-matched counterparts, despite sharing similar comorbidities, demography, sex, and age. Several studies suggest that inflammation affects immune-mediated pathways, multimorbidity, and frailty by inhibiting growth factors, increasing catabolism, and by disrupting homeostatic signaling. Frailty is more common in the community-dwelling population as people get older, ranging from 7 to 10% in those over 65 years up to 40% in those who are octogenarians. Different parameters have been validated to identify frailty. These primarily relate to two conceptual models: Fried’s physical frailty phenotype and Rockwood’s cumulative deficit method. Immune-mediated rheumatic diseases (IMRDs), such as rheumatoid arthritis, spondyloarthritis, systemic lupus erythematosus, systemic sclerosis, and vasculitis, are leading causes of frailty in developing countries. The aim of this review was to quantitatively synthesize published literature on the prevalence of frailty in IMRDs and to summarize current evidence on the relevance and applicability of the most widely used frailty screening tools.

## Frailty—from Concept to Rheumatology Clinical Practice

Frailty is a multidimensional condition which is defined as “a biologic syndrome of decreased reserve and resistance to stressors, resulting from cumulative declines across multiple physiologic systems, and causing vulnerability to adverse outcomes” [1]*.* In recent years, frailty has emerged as a significant area of research in rheumatology [2–5]. This condition has a deep impact on patient’s quality of life, morbidity, and longevity, as well as a considerable effect on medical and public spending costs. Nowadays, it is regarded as one of the main health-care problems [6, 7]. Beside frailty, pre-frailty is defined as a status that occurs before the onset of frailty and which is linked to the later onset of frailty. As a result, pre-frailty could be a more strategical target for screening and timing intervention [8].

**Fig. 1:**
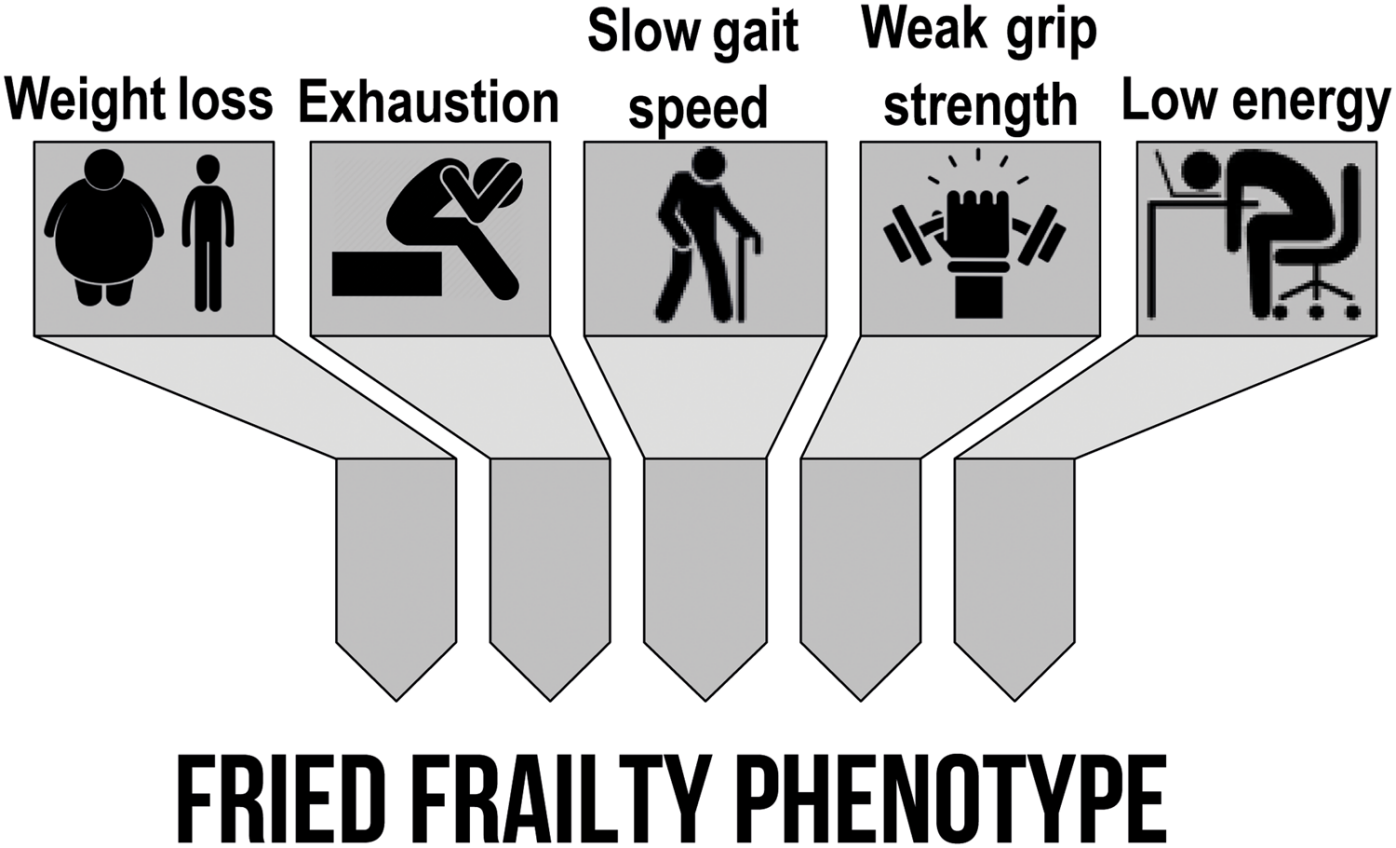
Criteria to identify frail subjects: the physical frailty phenotype proposed by Fried

In the literature, different parameters have been validated to classify frailty. Frailty belongs primarily to two conceptual models: Fried’s Physical Frailty phenotype [1] and Rockwood’s cumulative deficiency approach [9]. Both models have been scientifically validated. The first is based on the concept that, although each individual condition may be minor in and of itself, the frailty syndrome is created by the total number of individual dysfunctions and their interactions. Fried et al. [1] defined frailty as weight loss, fatigue (or exhaustion), slow gait speed, weakness (weak grip strength), and low levels of energy (or physical activity) in community-dwelling adults, geriatric medicine [10], but also in other specialties [11] (Fig. 1). Those who meet none of these criteria are classified as “robust,” whereas those who meet one or two criteria are classified as “pre-frail,” and those who meet three or more criteria are classified as “frail.”

Rockwood et al. described frailty as a complex, multidimensional condition characterized by the loss of reserves such as wealth, physical strength, intellect, and health, thereby increasing individual’s vulnerability [12]. In this model, defined as Frailty Index (FI), deficits are evaluated across multiple domains [9]. The term frailty is used to measure differences in susceptibility to negative outcomes [13]. Individuals with few deficits are regarded as fit, while those with a greater number of health conditions are considered as frailer and, as such, are more vulnerable to negative outcomes.

## The Emblem of Frailty: Geriatric Cohorts

The incidence of frailty in older geriatric populations is highly variable. Frailty affects 4–17% of the general population, mainly women (nearly twice as much as in men) and increases with age [1, 14, 15]. Many studies have focused on pre-frailty (i.e., patients at-risk for frailty but who do not fulfill all the criteria for being categorized as frail) and have reported a prevalence rate ranging from 28 to 44% [1]. Variations in frailty meanings and operationalizations, as well as the population surveyed, are possible explanations for this large variability. According to a recent literature systematic review and meta-analysis analyzing data from 22 European countries involved in the Joint Action initiative on Frailty (or ADVANTAGE), the prevalence of frailty in group and in non-community-based studies was 12% and 45%, respectively [16].

Based on Fried’s phenotype [1], the prevalence of susceptible individuals over 65 years was 9.9% for frailty and 44.2% for pre-frailty [14].

According to the Survey of Health, Ageing and Retirement in Europe (SHARE) cohort’s results, 12.1% of Germans over the age of 65 are frail [8]. The combined prevalence was 15% in ten population studies in Italy including patients aged 65 years or older. A close connection between frailty and psychosocial causes has been documented, meaning that both physical and psychosocial dimensions of human functioning should be considered [17]. Among classification systems, Fried’s frailty phenotype is the most known according to the findings of the Cardiovascular Health Study (CHS) [1].

Because of the broad range of inclusion criteria, the heterogeneity between studies is significant. This is a relevant aspect as no single consensus definition of frailty has been developed yet. In addition, the FI and the Frailty Phenotype, albeit complementary to each other, do not measure the same constructs.

## The Biological Basis: Sarcopenia and Immune Senescence (and Their Bidirectional Link)

Sarcopenia potentially represents the biological substrate of physical frailty. Sarcopenia is increasingly recognized as a correlate of ageing which is associated with increased likelihood of adverse outcomes including falls, fractures, frailty, and mortality [18, 19]. The European Working Group on Sarcopenia in Older People (EWSOG) defines sarcopenia as a syndrome characterized by progressive and generalized loss of skeletal muscle mass and strength, leading to increased adverse health outcomes such as falls, hospital admission, and mortality [20]. Physical inactivity, malnutrition, and elevated oxidative stress are all factors potentially involved in the pathogenesis of age-related sarcopenia. A recent meta-analysis including community dwelling, nursing home, and hospitalized patients over the age of 60 suggested a higher mortality risk (odds ratio 3.6) in individuals with sarcopenia [21]. While primary sarcopenia is considered as part of the normal ageing process, secondary sarcopenia has been described in conditions that are not solely a consequence of the ageing process. These include malabsorptive conditions, immobility/bed rest, starvation, hypothyroidism, osteoporosis, and several inflammatory conditions, such as immune-mediated rheumatic diseases (IMRDs) [22].

**Fig. 2:**
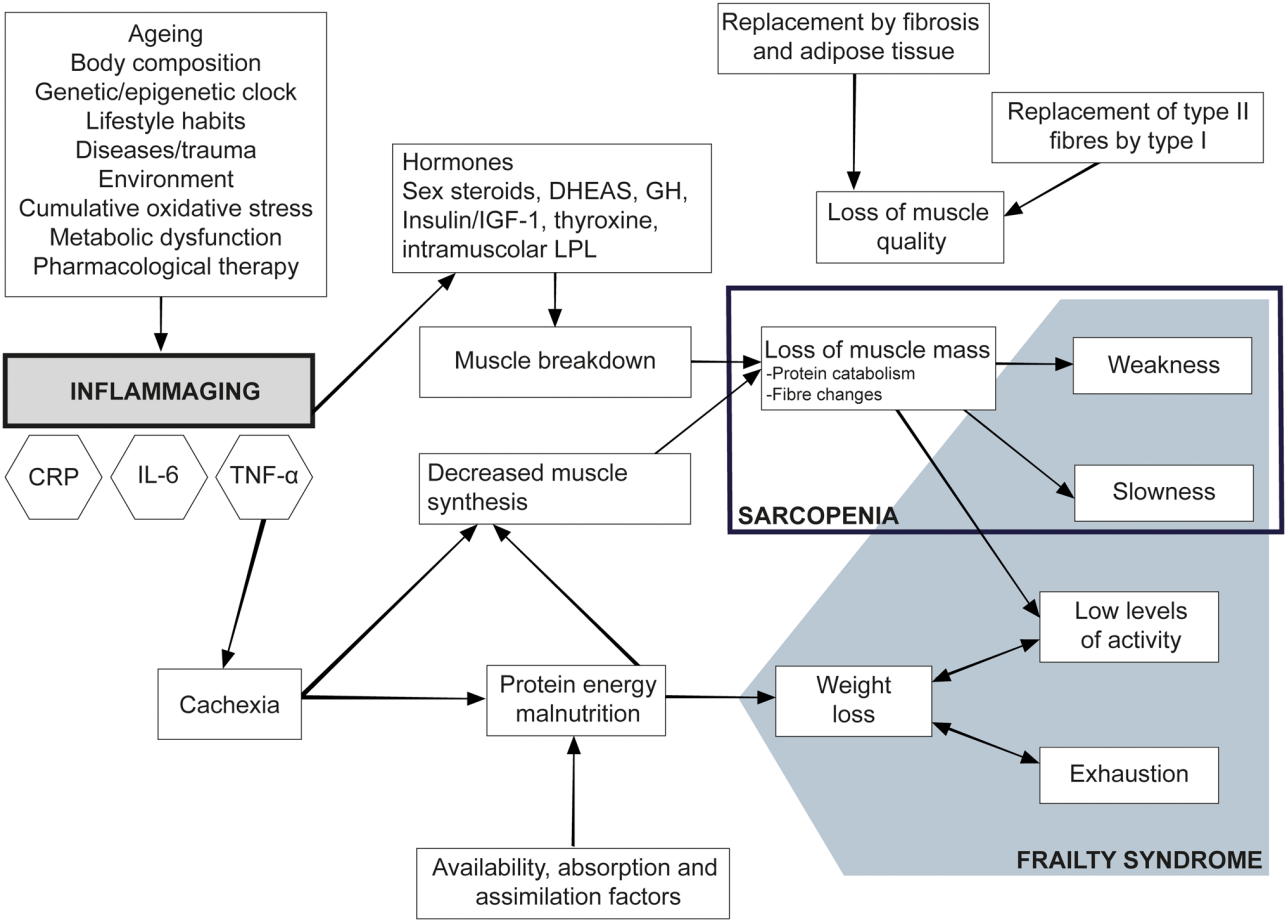
Relationship between inflammaging, sarcopenia, and frailty

The biological mechanisms responsible for sarcopenia are complex. Among the various factors involved in the aetiology of muscle weakness and sarcopenia, it seems clear that chronic inflammation plays a major role [23]. In fact, there is increasing evidence that raised inflammatory cytokines, possibly in combination with reduced growth factor levels, contributes to the development of sarcopenia and age-related physical decline [24]. Furthermore, epidemiological studies have shown that there is a correlation between high levels of inflammatory markers, such as interleukin (IL)-6 and C-reactive protein (CRP), low levels of insulin growth factor (IGF)-1, high levels of oxidative stress, decreased mitochondrial function, and muscle weakness [25] (Fig. 2). Alongside, a potential correlation between hormonal reduction and inflammation has been hypothesized, since estrogen decline seems to increase the levels of pro-inflammatory cytokines such as IL-6 and tumor necrosis factor (TNF)α [26].

## The Interplay Between Immunosenescence and Age-Related Musculoskeletal Diseases

Inflammation plays a very important role in rheumatic disorders, especially in rheumatoid arthritis (RA), and ageing’s pro-inflammatory state is a strong risk factor for many IMRDs. Immunosenescence, epigenetic clock, endothelial cell senescence, metabolic dysfunction, oxidative stress, sarcopenia, inactivity, malnutrition, and multimorbidity can all contribute to the loss of physical function and a global physical decline, resulting in frailty [27]. Chronic systemic inflammation is a common driver of age-related frailty (Fig. 2). A significant elevation in pro-inflammatory cytokines and of the inflammatory index among older adults who were pre-frail or frail has been described [28].

There are several theories explaining the possible connection between inflammation, ageing and frailty. Interestingly, some of the mechanisms sustaining this connection are those involved in the pathogenesis of IMRDs. The age-related upregulation of the inflammatory response is known as inflammaging [29]. Inflammaging is a pathological phenomenon and a key concept that connects the knowledge of ageing-related chronic illness, functional deterioration, and frailty throughout the lifespan. Ageing is linked to chronic inflammatory responses due to increased circulatory inflammatory cytokine production. Elevated serum levels of inflammatory markers (such as CRP), and pro-inflammatory cytokines (such as IL-6 and TNFα) have been linked to poor function, reduced mobility status, and sarcopenia [30, 31]. For example, the In the Invecchiare in Chianti (InCHIANTI) study (1020 men and women older than 65 years) showed a substantial correlation between inflammation (IL-6, IL-1R, and CRP levels) and both poor physical performance and decreased muscle strength [23]*.*

Studies comparing various inflammatory markers support the role of CRP in the etiology of frailty [32, 33]. Indeed, it has been proven that CRP levels significantly positively correlate with body mass index (BMI) and fat mass [34]. In a study involving patients with acquired immunity deficiency syndrome, high CRP levels showed a significant correlation with skeletal muscle loss [35]. In the Cardiovascular Health Study (CHS), patients were followed-up for nine years, and CRP was found to be an independent predictor of frailty [36]. Similarly, in the Longitudinal Aging Study Amsterdam (LASA), elevated CRP levels were associated with development of frailty at 3-year follow-up [33]. Sarcopenia has been reported in almost 30% of Japanese RA patients [37]. While advanced age, BMI, elevated levels of CRP, and decreased bone mass were all related to frailty in this population, CRP levels and muscle mass showed an inverse relationship [38].

IL-6 is a proinflammatory cytokine and an anti-inflammatory myokine which is produced by T cells, macrophages, fibroblasts, and endothelial cells. There are two distinct mechanisms of action for IL-6. The first is “normal” IL-6 signaling, which is primarily regenerative, defensive, and anti-inflammatory and involves membrane-bound receptors (IL-6R). The second pathway, which includes the soluble IL-6R (sIL-6R), is pro-inflammatory [39]. Age is associated with increased IL-6 gene expression, age-related diseases, and frailty [40, 41]. Higher levels of IL-6 (> 5 pg/mL) in the elderly have been related to an increased risk of muscle weakness and reduced handgrip strength [24, 34].

TNFα levels in the blood are significantly higher in the elderly than in the middle-age population, thus supporting the theory that inflammatory biomarkers rise with age [42]. In a study in older people, higher levels of TNFα were linked to a decrease in thigh muscle cross-sectional area and handgrip strength, implying that TNFα could be reliably linked to muscle mass and strength loss [43]. TNFα can induce muscle loss by promoting protein degradation and by decreasing protein synthesis [44]. TNFα elevation has also been shown to accelerate catabolic pathways in skeletal muscle [45]. The upregulation of TNFα is thought to cause muscle proteolysis, which results in muscle loss and sarcopenia. These combined factors can result in cachexia [46], a condition which is characterized by loss of muscle mass (i.e., muscle atrophy) and strength, changes in muscle fibers, increased inflammatory biomarkers in the muscle, and preserved (or increased) fat mass [47]. TNFα causes both type I and type II muscle fiber apoptosis [48], which may be one of the factors. TNFα also inhibits myogenic differentiation by destabilizing the myoblast determination (MyoD) protein [49] (Fig. 2).

## Frailty in the Immune-Mediated Rheumatic Diseases

Several cross-sectional studies have shown a higher prevalence of frailty in IMRDs, such as RA, psoriatic arthritis (PsA), ankylosing spondylitis (AS), systemic lupus erythematosus (SLE), systemic sclerosis (SSc) and vasculitis [2, 50–53].

### Frailty in Rheumatoid Arthritis

RA is a systemic autoimmune disease characterized by chronic synovial joint inflammation that causes cartilage loss, bone degradation, and joint integrity impairment, leading to reduced patient’s physical function and health-related quality of life (HRQoL) [54, 55].

There have been several signs of premature immunosenescence in RA patients, such as reduced thymic functionality, expansion of late-differentiated effector T cells, increased telomeric attrition, and increased development of pro-inflammatory cytokines (senescence-associated secretory phenotype) [56]. A significant expansion of late differentiated T cells (CD4 + CD28 − and CD8 + CD28 −) has been described in RA [57]*,* similarly to what has been observed in healthy ageing [58]. Immunosenescence seems to play a crucial role in the development of RA comorbidities since both RA and comordibities are immune-mediated. RA-related comorbidities include cancer, cardiovascular disease, respiratory disease, osteoporosis, cognitive impairment, and depression [59]. Cognitive decline is a psychophysical disorder characterized by changes in orientation, attention, problem-solving ability, memory, and executive functions [60]. Both depression and frailty are usually associated with high levels of pain and disability, low HRQoL, and increased mortality [10, 61–63]. Depression can also be a predictor of frailty, predisposing to reduced social ties, gait speed, and physical activities, and to an increased sedentary behavior, fall risk, weight loss, and malnutrition, all of which may contribute to the perpetuation of depressive symptoms (i.e., sadness, anhedonia, and helplessness) [64]. Depression and anxiety affect 66% and 70% of RA patients, respectively [65, 66]. In addition, almost 20% of RA patients is affected by a major depressive disorder. Depression has been linked not only to physical frailty and social interactions, but also to cognitive disability [67]. Unlike RA patients in clinical remission, those with an active disease have a worsened cognitive function [68]. A variety of factors may be responsible for cognitive disorders in RA. The initiating factor may be the systemic inflammation related to the chronic rheumatological condition [69, 70]. Autoantibodies, such as rheumatoid factor (RF) [71], immune complexes, and cytokines, can trigger neuroinflammatory responses in the brain [72]. It has been proposed that cytokines, such as IL-1 and TNFα, can modulate neuron excitability through non-canonical signaling pathways, as well as interactions with receptors [73]*.* Furthermore, a detrimental effect of IL-6 on cognition has been described [74]. Another possible cause of cognitive dysfunction in RA may be the expansion of senescent cells. A negative correlation between the expansion of late-differentiated CD8 + CD28 T cells and memory function has been found in RA patients [57]. On the other hand, patients with a higher number of memory T cells (CD45RO +) had greater cognitive abilities. Another potential immune-related cause for impaired cognition in RA is the production of autoantibodies against brain antigens. With healthy ageing, there is a rise in the levels of circulating autoantibodies, which can be seen early in the disease course of RA. Consequently, immunosenescence and changes in cognition tend to have a synergistic effect and reduce life expectancy in the elderly [57]. Despite improvements in the therapeutic armamentarium, people with RA still suffer from physical disability and reduced HRQoL, and a more comprehensive understanding of the possible factors that lead to frailty in RA is critical.

### Frailty in Ankylosing Spondylitis and Psoriatic Arthritis

Ankylosing spondylitis (AS) and psoriatic arthritis (PsA) are part of the family of seronegative spondyloarthropathies. AS usually affects patients younger than 30 years old and it is associated with the HLA-B27 antigen. Patients with AS suffer from ossification of spinal ligaments, discs, endplates, and apophyseal structures giving the vertebral column the classic “bamboo spine” appearance in the late stages. Kyphotic deformity, an ankylosis of the craniocervical junction, can also be observed in AS [75]. PsA affects men and women equally, usually after age 30. Psoriasis and PsA both run in families; they are more common in whites than in other races/ethnicities. The prevalence of PsA varies from 20 to 420 per 100,000 population across the world except in Japan where it is 1 per 100,000 [76]. PsA articular involvement is extremely heterogeneous. It has been classified into five main subtypes (i.e., distal interphalangeal joints predominant, symmetrical polyarthritis, asymmetrical oligoarthritis and monoarthritis, predominant spondylitis, and arthritis mutilans).

The paucity of studies focusing on frailty in patients with spondyloarthritis is striking if compared to patients with other inflammatory joint diseases, such as RA. Frailty seems less common in AS/PsA than in RA, but after controlling for confounders, the prevalence of frailty appears to be similar in both diseases. Reduced bone mass, stiffness, and movement loss are commonly observed in AS; these can be linked to muscle loss and, therefore, the development of sarcopenia. The incidence and effect of sarcopenia in AS patients, however, has yet to be determined [77]. In male (but not in female) patients with AS, a decrease in muscle mass (and fat mass) is correlated with higher disease activity [78]. In a small cross-sectional study, a substantially lower appendicular lean body mass (but no total mass), lower muscle strength, and a decreased number of type II muscle fibers was found in patients with AS in comparison with healthy controls [79]. Similarly, a lower muscle mass was found in 67 males with AS in comparison with healthy controls. Pre-sarcopenia, sarcopenia (as described by EWGSOP), and cachexia were found in 50.4%, 34.3%, and 11.9% of patients with AS, respectively. Higher disease activity and lower bone mass density were associated with sarcopenia and cachexia [80]. The prevalence of sarcopenia in patients with AS was high (around 20%) in another cross-sectional study [77]. Like RA, frailty in spondyloarthritis may also be caused by cognitive impairment. Mild cognitive impairment has been observed in around half of PsA patients, and lower cognitive performance has been linked to age, physical handicap, and weariness [81]*.*

### Frailty in Systemic Lupus Erythematosus

SLE is considered as a disorder that affects women during their reproductive years. However, it is known that SLE can also affect people over the age of 50 [82, 83].

Several studies have investigated the prevalence, clinical relevance, and prognostic impact on disease outcomes of frailty in patients with SLE. In a study assessing frailty in 152 women with SLE using Fried’s frailty criteria, around 20% of patients was classified as “frail” (≥ 3 Fried’s criteria met) and 50% as “pre-frail” (1 or 2 Fried’s criteria met). Exhaustion, weakness, and physical inactivity were the most prevalent components of frailty, being reported in 45.4%, 30.9%, and 29.0% of the patients, respectively. Of note, frail patients were more likely to have worse physical function and cognitive impairment in comparison with non-frail patients. In addition, mortality risk was almost six time higher for patients classified as “frail” than for patients classified as “robust” (i.e., no Fried’s criteria met) [52]. In 2019, a secondary analysis of longitudinal data from the Systemic Lupus International Collaborating Clinics (SLICC) led to the construction of a frailty index (FI) as a measure of vulnerability to adverse outcomes in patients with SLE [84]. The SLICC-FI includes 48 different health deficits, of which 14 are related to organ damage (i.e., heart failure or chronic kidney disease), 14 reflect ‘active’ inflammation (e.g., arthritis or serositis), 6 are related to comorbidities (e.g., high blood pressure or obesity), and the remaining 14 to patient’s function, mobility, health attitude, and mental health. In this study, 452 out 1682 (27.1%) SLE patients were considered as “frail” based on SLICC-FI values > 0.21. Older age, female sex, lower education, and cigarette smoking were more prevalent in frail patients than in relatively fit patients. Interestingly, almost 20% of SLE patients < 30 years old were classified as frail. This prevalence appears to be high if compared to the estimated prevalence of frailty (around 2.0%) among the general population in the same age group [85]. The validity and clinical impact of the SLICC-FI was subsequently evaluated by the SLICC group in different cohorts of SLE patients. SLICC-FI was found to be predictive of mortality risk [86], damage accrual [87], and hospitalization among patients with SLE. These results confirmed the value and utility of SLICC-FI as a measure of general health in SLE. Some studies also reported on single elements of the frailty phenotypes (i.e., sarcopenia, muscle strength) in patients with SLE. Muscle strength (i.e., grip strength and 1-kg arm lift) and physical performances (i.e., 30-s sit to stand, knee extension, hip flexion, hip abduction, and shoulder flexion) are significantly reduced in SLE patients in comparison with healthy controls [88]. Another study documented a significant association between reduced lower limb muscle strength, evaluated by peak knee torque of extension and flexion and by chair‐stand time, and physical disability scores [i.e., SF‐36 and Valued Life Activities (VLA) disability scores] in 146 SLE women from the Lupus Outcome Study cohort [89]. A study on body composition revealed the presence of sarcopenia (defined as fat-free mass index on dual-energy X-ray absorptiometry) in around 20% of SLE patients but only in 6.5% of controls [90]. Compared with the control group, SLE patients were less likely to have normal body composition, despite having a similar BMI. In addition, 6.5% of SLE patients but no controls was classified as having sarcopenic obesity (*p* = 0.03).

### Frailty and Systemic Sclerosis

Only a very few studies investigated frailty in patients with SSc. In 2013, the Canadian Scleroderma Research Group (CSRG) developed and validated a FI (including 41 items) for patients with SSc [91]. The CSRG-FI was significantly correlated with the Rodnan Skin Score (r = 0.28 in SSc patients with diffuse disease; r = 0.18 in patients with limited disease) and, to a lesser extent, with the physician assessment of damage (r = 0.51 for both limited and diffuse disease). In addition, the authors found an association between higher FI scores and the risk of death (HR 1.21). The relationship between interstitial lung disease (ILD) and frailty in SSc has also been investigated. Frailty was found to be prevalent in 55% of patients with SSc-ILD and to be strongly correlated with dyspnea when measured using a 42-item index. Even though SSc patients had a significantly younger age, the FI did not differ significantly from that of a control population with ILD not associated with a connective tissue disease, indicating that chronological age significantly underestimates biological age in SSc patients and that the concept of frailty could enable a more accurate prognostic evaluation than demographic and disease-related factors. Patients with SSc and ILD (*n* = 86) and patients with non-systemic (i.e., without connective tissue disease) ILD sub-types (*n* = 186 patients) had similar mean scores on a 42-item FI; interestingly, dyspnea had the highest degree of association with frailty (r = 0.62, *p* < 0.001) and was the only variable independently associated with frailty on multivariable analysis [53]. Sarcopenia was found to be prevalent in a large cohort of 141 patients with SSc, with a prevalence of about 20.7%. Sarcopenia prevalence was substantially higher in malnourished patients [92]. After a 6-week dietary intervention, the percentage of patients with sarcopenia (defined by muscle mass) was lower (54–39%, *p* = 0.02) in a pilot intervention study of 18 patients with SSc and gastrointestinal involvement. In patients with SSc, however, no connection between sarcopenia and disease duration was identified [93].

These studies reveal an intriguing link between IMRDs and sarcopenia, but they are hampered by differences in sarcopenia-related term definitions, especially those that only assess muscle mass versus those that use a modern definition.

### Frailty in ANCA-Associated Vasculitis

Antineutrophil cytoplasmic antibodies (ANCA)-associated vasculitis (AAV) is a group of necrotizing vasculitis, characteristically associated with the presence of serum ANCA, that primarily affects small vessels of the body. Lung involvement, and pauci-immune crescentic glomerulonephritis, which may contribute to end-stage renal disease, can be observed. AAV is thought to be present in about 100 people per million. Survival rates after five years vary from 45 to 97%. High-dose glucocorticoids and immunosuppressive drugs, such as cyclophosphamide or rituximab, are used to induce remission in severe cases with lung or kidney involvement. While the available treatments for AAV are useful to suppress chronic inflammation (thus leading to increased survival), they can lead in the long term to sarcopenia, bone loss, increased infection risk, and frailty [94]. A recent study evaluated the prognostic impact of frailty (scored using the Rockwood’s Clinical Frailty Scale) on the long-term outcomes in 83 elderly patients (≥ 65 years old) with AAV. Frailty scores (HR 1.90), age (HR 1.13), and very high baseline CRP values (HR 5.71) were independently associated with increased risk of death [95]. In addition, adverse events and hospitalization were significantly higher in the frailer group in comparison with the less frail group (*p* = 0.065 and *p* = 0.02, respectively). Patients with a lower vs. higher baseline frailty score (score three vs. score four) had no variations in time to recovery or relapse, but the more frail community had a higher proportion of adverse events, such as longer hospitalization and mortality, with a five-year survival rate of 47 vs. 90%.

## Frailty Screening Tools

Several instruments have been developed to measure frailty as part of a stepwise assessment of vulnerability. To classify older people as frail, different criteria have been validated in the literature, which mainly apply to two conceptual models: Fried’s phenotype of physical frailty [1] (Fig. 1) and Rockwood’s cumulative “index process” [9, 12]. These two instruments are very different, and they should be considered complementary rather than competitive. Both models have been empirically tested, but there is no agreement on which of the two should be used to assess frailty.

### Fried’s Frailty Phenotype

Fried’s frailty phenotype is a popular measurement of frailty [1] (Fig. 1). It is based on the collection of five pre-defined parameters that examine the presence/absence of the following signs and symptoms: involuntary weight loss, exhaustion, slow gait speed, poor handgrip strength, and sedentary behavior. The number of criteria (a 6-level ordinal variable ranging from 0 to 5) is divided into three categories: robustness (none), pre-frailty (one or two criteria), and frailty (three or more criteria). The parameters refer to (1) weight loss: unintentional weight loss of more than 10 pounds in the previous year; (2) exhaustion: participants reporting that all they did was an effort or that they could not get moving a moderate amount of the time or much of the time [from the Center for Epidemiological Studies-Depression (CES-D) Scale]; (3) physical activity (Minnesota Leisure Time Activity Questionnaire): energy consumption of 383 kcal per week for men and 270 kcal per week for women; (4) grip strength (Jamar Dynamometer, Layfayette Instruments, USA) (average of three trials): 29–32 kg for men (stratified by BMI classifications) and 17–21 kg for women (stratified by BMI classifications); (5) walk time (15-ft walk): 7 s (men height 173 cm, women height 159 cm) or 6 s (men height > 173 cm, women height > 159 cm).

### Survey of Health, Ageing and Retirement in Europe Frailty Instrument

A phenotypic approach to frailty is that of the Survey of Health, Ageing and Retirement in Europe Frailty Instrument (SHARE-FI). Indeed, while Fried’s phenotype is based on the number of criteria met, the frailty score and cut-offs for defining the frailty classes (non-frail, pre-frail, and frail) in SHARE-FI are based on latent variable modeling [96]. The variables assessing frailty in SHARE-FI are explored as follows [8]. “Have you had too little energy to do the things you wanted to do in the last month?” is used to identify fatigue. A positive response is coded as 1, while a negative response is coded as 0. “How has your appetite been?” explores the weight loss criteria, defined by reporting a “Diminution of desire for food” or by answering “Less” to the question: “So, have you been eating more or less than usual?” in the case of a non-specific or uncodeable answer to this question. The presence of the criterion is coded as 1, and the absence of the criterion is coded as 0. Handgrip strength is measured using a grip system to determine weakness. Slowness is described as a positive response to either one of the following two questions: “Do you have difficulty [expected to last more than 3 months] walking 100 m because of a health problem?” and “Do you have difficulty [expected to last more than 3 months] walking 100 m because of a health problem?” or “…climbing a flight of stairs without taking a break?”. Each positive response gives a score of 1. The question “How often do you participate in activities that involve a low or moderate amount of energy, such as gardening, cleaning the car, or going for a walk?” was used to assess the low activity criterion. This variable was held ordinal, with 1 indicating “more than once a week,” 2 indicating “once a week,” 3 indicating “one or three times a month,” and 4 indicating “rarely or never.” The SHARE-FI is calculated using the parameters mentioned above, and its calculators (one for each gender) are freely available online (http://www.biomedcentral.com/1471-2318/10/57/additional, translated versions are available at https://sites.google.com/a/tcd.ie/share-frailtyinstrument-calculators/). The calculator produces a continuous frailty score (i.e., the predicted discrete factor score, whose formulae are included in the paper) as data is entered and allows for automatic classification into three phenotypic frailty categories: non-frail, prefrail, and frail. SHARE-FI has the potential to enhance primary care quality by offering a quick and reliable way to assess and monitor frailty in community-dwelling people over 50, as well as a novel auditing and testing process.

### The Frailty Index of Cumulative Deficits

The FI of cumulative deficits (FI-CD) was first proposed by Rockwood and Mitnitski as a way to incorporate the multidimensional nature of frailty into an operational definition [97]. A long list of chronic disorders and illnesses is part of the FI-CD. Risk assessments have been stated to be reliable when a minimum of 50 items are considered, but shorter versions (as few as 20 conditions) have also been proposed. Although the FI-CD has been classified to assess dichotomous conditions (e.g., robustness vs. frailty), its main distinguishing feature is its continuous existence. The FI-CD is obviously not applicable at the first patient assessment because it can only be generated after (or in parallel with) a detailed geriatric evaluation. The FI-CD becomes highly informative for the subject’s follow-up. In particular, the FI-CD phenotype is likely to be more susceptible to changes than the categorical frailty phenotype. As a result, the FI-CD could be a better method of choice for determining the efficacy of any intervention and describing health condition trajectories over time. Despite its many advantages, the FI-CD has some drawbacks: it can be time consuming, and its mathematical nature, though basic, makes it unpopular in clinical settings. Among the FI-CD instruments are included the Groningen Frailty Indicator (GFI) [98], the Edmonton Frail Scale (EFS) [99], the Comprehensive Geriatric Assessment (CGA) [100], the Tilburg Frailty Indicator (TFI) [101], the PRISMA-7 questionnaire [102]*,* and QFrailty score [6]. Recently, our group has developed and preliminarly validated way a tool to assess frailty, dedicated to RA patients and easy to use in clinical practice, the Comprehensive Rheumatologic Assessment of Frailty (CRAF) [103].

### Groningen Frailty Indicator

The Groningen Frailty Indicator (GFI) is a widely used instrument to measure frailty, which has been developed in the Netherlands, with moderate internal consistency and adequate discriminative ability [98]. It includes 15 dichotomous self-reported products, including physical factors (independence in shopping, walking, dressing, and toileting; physical fitness, vision, hearing; weight loss, and polypharmacy); a cognitive component (memory issues); social factors (emptiness, missing others, and feeling abandoned); and a psychological component (depression) (feeling downhearted or sad; feeling nervous or anxious). GFI defines frailty as a score ranging from 0 (normal behavior without restriction) to 15 (completely disabled), with scores of 4 indicating frailty [104]. As a frailty measurement, the GFI shows good feasibility and reliability.

### Edmonton Frail Scale

The Edmonton Frail Scale (EFS) is a valid and reliable measurement tool for the identification of frailty in the hospital setting [105]. The EFS is a 17-point scale that measures memory, general health, self-reported health, functional independence, social support, polypharmacy, mood, continence, and functional efficiency [99]. The number of the component scores is used to classify frailty severity, with the following cut-off scores: not weak (0–5), vulnerable (6–7), slightly frail (8–9), moderately frail (10–11), and extremely frail (12–17). A frailty state is assigned to patients with a score of 8 or greater [106]. In a community-based sample, it was a valid measure compared to the clinical judegment of geriatric specialists [105]. The EFS has been shown to predict complications and negative outcomes in elderly patients who are having elective surgery and who are admitted to the hospital with acute coronary syndrome [107].

### Comprehensive Geriatric Assessment

The Comprehensive Geriatric Assessment (CGA) is a multidisciplinary and multidimensional tool [100]. CGA is used as both a screening method and for designing treatment plans. CGA is a comprehensive interdisciplinary system for the evaluation of the functional status of the elderly with the final objective to establish a coordinated plan to improve their overall health. It contains 15 questions that are divided into three categories: functional status [seven questions on activities of daily living (ADL) and instrumentalADL), cognitive status [four questions from the Mini Mental State examination (MMSE)], and depression (four questions from the Geriatric Depression Scale (GDS)-15). Scores of > = 1 for ADL and IADL, 6 for the MMSE, and 2 for the GDS-4 were defined as cut-off values to indicate whether a more detailed evaluation was needed. The results of these assessments are used to develop a management plan that incorporates realistic treatment goals for both the patient and caregiver. If a positive score is found in one of the CGA domains, further frailty assessment is required [108].

### Tilburg Frailty Indicator

The Tilburg Frailty Indicator (TFI) is a self-administered questionnaire that was created in 2010 in the Netherlands [101]. TFI assesses frailty on three levels. The physical domain assesses physical fitness, weight loss, walking, balance, hearing, vision, hand strength, and tiredness. The psychological area includes memory, depression, anxiety, or nervousness, and problem-solving. In the social domain, factors such as living alone, missing people, and not getting enough treatment are considered. The total score of the TFI is calculated by giving a score to each item, yielding a total score that ranges from 0 to 15. The physical domain ranges from 0 to 8, the psychological domain from 0 to 4, and the social domain from 0 to 3. Scores of 5 out of 15 indicate frailty. For community-dwelling older people, the TFI has good validity and reliability. In contrast to its social components, the TFI’s physical components have been found to have a high predictive value for negative outcomes.

### PRISMA-7 Questionnaire

The PRISMA-7 questionnaire was realized during the Program on Research for Integrating Services for the Maintenance of Autonomy (PRISMA Project) in 2007 [102]. It is a three-minute French-language self-administered questionnaire. Seven basic objects are used to investigate sex, autonomy, close circle, and walking. The validation sample consisted of 594 people aged 75 and up who were randomly chosen from electoral lists. The SMAF scale (Système de Mesure de l'Autonomie Fonctionnelle), a 29-item scale, was then used to give each participant a geriatric assessment [109].

### QFrailty Score

The National Institute for Clinical Excellence (NICE) guidance on multiple morbidities highlighted the need for new rigorous equations to identify patients in primary care with reduced life expectancy [110]. To identify frailty-prone patients, a new algorithm (QFrailty score) was developed and externally validated [6]. The QFrailty score is based on data obtained from tens of thousands of general practitioners around the United Kingdom (UK) who have voluntarily contributed data to the QResearch database for medical research. When paired with the QAdmissions equation, the equation can be used to categorize patients into four QFrailty groups, allowing for more tailored assessments and treatments. Based on predicted mortality risks and unplanned hospital admissions, 2.7% of patients were classified as seriously fragile, 9.4% as moderately frail, 43.1% as slightly frail, and 44.8% as fit. The electronic frailty index (EFI) (https://qfrailty.org) is a simple unweighted count of a patient’s total number of “deficits” out of 36, where a deficit is described as a physical deficiency or social weakness as decided by a consensus panel. The EFI was also used to estimate mortality in a community-based population in the UK (using traditional definitions) [111, 112].

### Comprehensive Rheumatologic Assessment of Frailty Index

**Fig. 3:**
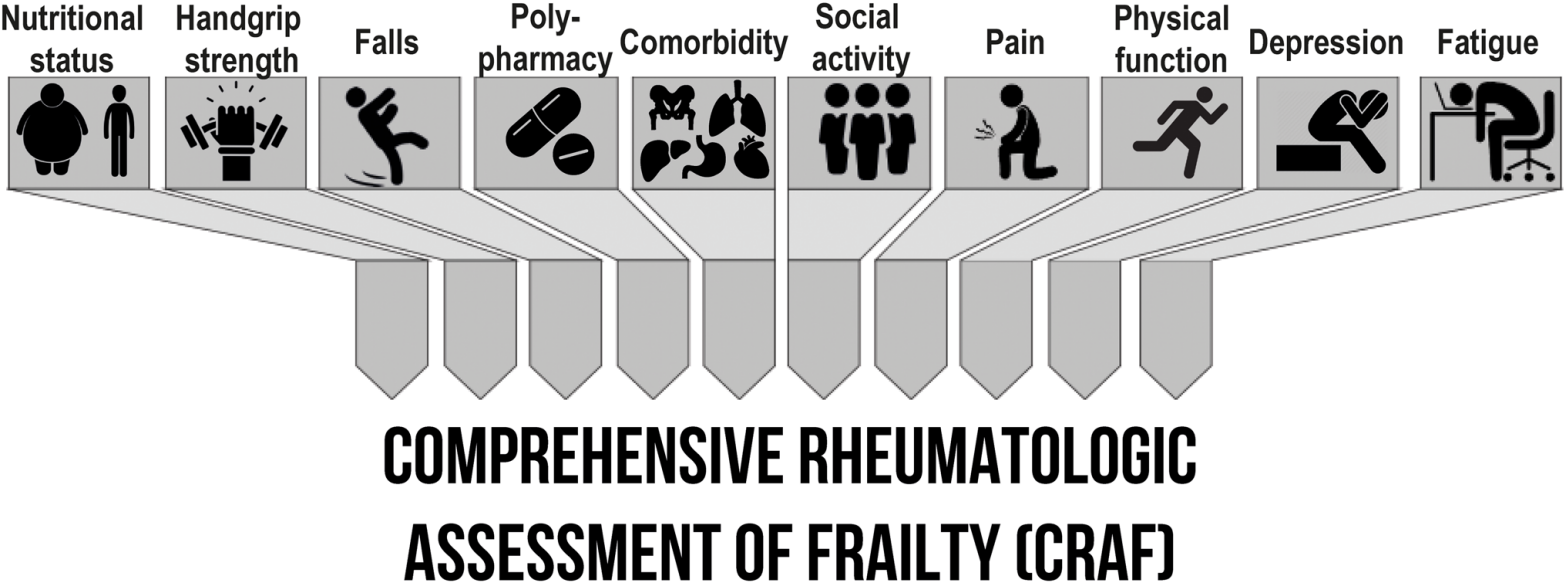
Domains evaluated in CRAF index

Similar to the FI created by Rockwood and colleagues [12, 96, 113], the Comprehensive Rheumatologic Assessment of Frailty (CRAF) index is calculated using accumulated deficits [103]. The CRAF index incorporates evidence from current clinical records and it can be used in rheumatology practice. A Delphi method was used to create the variables in the CRAF. Nutritional status, weakness, falls, comorbidity, polypharmacy, social activity, pain, exhaustion, physical function, and depression were defined as ten major frailty domains of CRAF (Fig. 3). The authors studied and selected 34 indicators from existing frailty appraisal tools based on the Gobbens frailty theory model [114]. Using Lynn’s process for content validation, 39 experts (19 rheumatologists, 6 rehabilitation medicine physicians, 6 geriatricians, 3 ortopaedist, 6 neurologists, and 2 internal medicine specialists) were asked to rate the importance of each variable in the measurement of frailty in RA patients. On a Likert scale from one to four, the importance of each variable was rated as follows: 1 = irrelevant, 2 = slightly relevant, 3 = relevant, and 4 = highly relevant. To be included in the CRAF, the variables had to obtain a mean score of > 3.0 (“extremely relevant”) from more than 80% of the expert group. The experts observed that nutritional status (measured by the BMI), weakness, falls, comorbidity, polypharmacy, social activity, pain, exhaustion, physical function, and depression were the factors most strongly associated with the likelihood of frailty in RA patients (Table 1). Handgrip strength was measured twice using an electronic grip device to determine weakness. The measurement of hand grip strength has been proposed as a biomarker of general health status and as an indicator of overall muscular strength [115]. According to the available data for grip strength, a T-score of − 2 (equal to 19 kg in females and 32 kg in males, or weaker) could define weakness in the general population, whereas a higher cut-off, a T-score of − 2.5 (corresponding to 16 kg in females and 27 kg in males), should be reserved to patients with coexisting osteoporosis [116]. In addition, the number of falls in the previous six months, a prominent indicator of instability in RA patients [117], was recorded asking the following question: “Have you had a fall in the last six months?”. The Rheumatic Diseases Comorbidity Index (RDCI) was used to determine the comorbidity load [118, 119]. The concurrent use of five or more drugs was defined as polypharmacy. Polypharmacy has been associated with a variety of negative outcomes, such as frailty, mortality, falls, adverse reactions to medications, longer hospitalization, and re-admission to the hospital shortly after discharge [120]. Social activities were also assessed using a semiquantitative scale (0–0.5–1). Patients were asked: “To what extent has your physical health or emotional problems interfered with your typical social activities with family, friends, neighbors, or groups during the previous 4 weeks?”.

**Table 1:** Deficits included in the Comprehensive Rheumatologic Assessment of Frailty (CRAF)

Concept/variable	Description	CRAF value
1. Nutritional status	Normal/overweight: BMI 25–30 kg/m^2^ Obese: BMI ≥ 30 kg/m^2^ Underweight: BMI < 18.5 kg/m^2^	0.00 0.50 1.00
2. Weakness	*Handgrip strength (in Kg) in men* < 27 T-score − 2.5 or below < 32 T-score − 2 or below > 32 Normal Grip *Handgrip strength (in Kg) in women* < 16 T-score − 2.5 or below < 19 T-score − 2 or below > 19 Normal Grip	1.00 0.50 0.00 1.00 0.50 0.00
3. Falls	Falls less than twice in the last 12 months Falls between two and five times in the last 12 months Falls more than five times in the last 12 months	0.00 0.50 1.00
4. Comorbidity	RDCI score 0 – 1 RDCI score 2 – 3 RDCI score 3 – 4 RDCI score 5 – 6 RDCI score ≥ 7	0.00 0.25 0.50 0.75 1.00
5. Polypharmacy	Patient uses less than two different medications Patient uses between three and four different medications Patient uses 5 or more different medications	0.00 0.50 1.00
6. Social activity	Not at all/slightly Moderately Quite a bit/extremely	0.00 0.50 1.00
7. Pain	Extreme pain Very severe pain Severe pain Moderate pain Mild pain No pain	1.00 0.80 0.60 0.40 0.20 0.00
8. Fatigue	Extreme fatigue Very severe fatigue Severe fatigue Moderate fatigue Mild fatigue No fatigue	1.00 0.80 0.60 0.40 0.20 0.00
9. Physical function	Extreme limitation Very severe limitation Severe limitation Moderate limitation Mild limitation No limitation	1.00 0.80 0.60 0.40 0.20 0.00
10. Depression	Extreme depression Very severe depression Severe depression Moderate depression Mild depression No depression	1.00 0.80 0.60 0.40 0.20 0.00

*RDCI* Rheumatic Disease Comorbidity Index, *BMI* Body Mass Index

*CRAF score of 0 to 0.12 = non frailty; > 0.12 to ≤ 0.24 = mild frailty; > 0.24 to ≤ 0.36 = moderate frailty; > 0.36 = severe frailty

Patients and experts agree that pain, weariness, physical function, and depression are all factors that contribute to frailty [121]. This is also ackwnoledged by both the Outcome Measures in Rheumatology (OMERACT) Patient Perspective Workshop and the American College of Rheumatologists (ACR)/European League Against Rheumatism classification criteria (EULAR) [122, 123]. Therefore, these 4 variables were included in the CRAF and scored using a six-level semiquantitative scale (0, 02, 0.4, 0.6, 0.8, and 1). The final score of CRAF is calculated by dividing the sum of the ten variables by 10: the score ranges from 0.0 (no deficit present) to 1.0 (all deficits present). Clegg’s criteria were used to determine the CRAF cut-off points, which are as follows: 0.12 = no frailty; 0.24 = mild frailty; 0.26 = moderate frailty; 0.36 = severe frailty [111]. The CRAF index is a comprehensive and promising tool for the assessment of frailty in patients with IMRDs. Further research is needed to explore its value and clinical impact in patients affected by RA, PsA, AS, and connective tissue diseases.

Table 2 summarizes the applications in single IMRDs of the various screening tools mentioned in this review.

**Table 2:** Application of screening tools for frailty in different IMRDs

Fried’s phenotype of physical disability	
Frailty Index (FI)	SLE, SSc
Survey of Health, Ageing and Retirement in Europe Frailty Instrument (SHARE-FI)	RA
Rockwood cumulative deficit model	
The Frailty index of cumulative deficits (FI-CD)	AAV
The Groningen Frailty Indicator (GFI)	RA
The Edmonton Frail Scale (EFS)	RA
Comprehensive Geriatric Assessment (CGA)	RA
Tilburg Frailty Indicator (TFI)	Never tested in IMRDs cohorts
Program on Research for Integrating Services for the Maintenance of Autonomy (PRISMA-7)	Never tested in IMRDs cohorts
QFrailty score	Never tested in IMRDs cohorts
Comprehensive Rheumatologic Assessment of Frailty (CRAF) index	RA

*SLE* systemic lupus erythematosus, *SSc* systemc sclerosis, *RA* rheumatoid arthritis, *AAV* ANCA-associated vasculitis, *IMRDs* immune-mediated rheumatic diseases

## Management of Sarcopenia

Management of sarcopenia should primarily be patient centered and involve the combination of both resistance and endurance based activity programmes with or without dietary interventions [124, 125]. Physical activity interventions and progressive resistance training have been suggested to have a predominant effect on muscle strength, muscle mass, and physical performance in older people [126]. Nutrition also plays a crucial impact in the course and clinical consequences of inflammatory illnesses like RA. Nutrition is increasingly being linked to muscle mass, strength, and function, implying that it plays a significant role in both the prevention and treatment of sarcopenia [127]. A systematic literature review looked at the link between sarcopenia and nutritional status and found that there was a link between sarcopenia and poor nutrition [128]. Unfavorable nutritional risk assessment results, insufficient protein, vitamin D, antioxidant nutrients, and long-chain polyunsaturated fatty acids intake, and sarcopenia have all been linked. Currently, no drug is registered for the treatment of sarcopenia. However, using particular inhibitors and/or medicines that affect epigenetics to manipulate the Janus kinase (JAK)/signal transducer and activator of transcription (STAT) (JAK/STAT) signaling system could be a promising therapeutic option for RA [129]. The JAK/STAT pathway is now widely regarded as being needed for successful muscle fiber adaptation during development and regeneration via IL-6 family signals. Through histone methylation and histone acetylation processes, the production of IL-6 and essential components of the JAK/STAT pathway is controlled at the epigenetic level. In addition, multiple studies have shown that the JAK/ STAT pathway is involved in controlling the myogenic development of adult satellite cells, a kind of cell that is critical for skeletal muscle postnatal growth and damage repair [130, 131].

## Conclusions and Perspectives

Several systematic reviews and published studies on frailty in IMRD patients have shed light on the overlap between frailty and musculoskeletal disorders, including potential pathogenic pathways and proposed therapies to prevent or reduce frailty. However, the general prevalence and understanding of factors that influence frailty in IMRDs have been inconsistently reported across research, and previous narrative reviews have not effectively synthesized this information. The need to strengthen frailty interventions and include vulnerable patients in future drug effects clinical trials is now understood, making the development of evidence-based guidelines far simpler. There are still a lot of unanswered questions that need to be addressed. The inclusion of components in the frailty group is a contentious issue with far-reaching implications. While some authors consider disability and functional decline to be an aspect of frailty [12, 13], others consider disability and functional decline to be an outcome. We believe that measuring frailty based on deficit accumulation provides a systematic approach to prognosis in IMDR patients, integrating disease function, organ damage, and HRQoL into a single measure. The CRAF index was created and validated to address this problem.

## Data Availability

Not applicable.
